# Does the ChOLE classification relate to the duration of surgery?

**DOI:** 10.1007/s00405-024-08997-z

**Published:** 2024-10-04

**Authors:** Julia Esser, Leonie Broicher, Marcel Mayer, Hans Eckel, Louis Jansen, Anne Nobis, Frederik Faste, Jens Peter Klussmann, Jan Christoffer Luers

**Affiliations:** Medical Faculty, Department of Otorhinolaryngology, Head and Neck Surgery, University of Cologne, 50924 Cologne, Germany

**Keywords:** Cholesteatoma, ChOLE classification, Duration of surgery, Cholesteatoma, Tympanoplasty

## Abstract

**Introduction:**

Cholesteatoma, a challenging entity in otologic surgery, necessitates a standardized classification system for effective communication among healthcare providers and consistent reporting of surgical outcomes. The ChOLE Classification System, introduced by Linder et al., stages cholesteatoma based on extension (Ch), ossicular chain status (O), life-threatening complications (L), and Eustachian tube function and mastoid pneumatization (E).

**Methods:**

We classified 199 patients who underwent cholesteatoma surgery between 2019 and 2023 in our University Hospital to assess the distribution of the ChOLE stages and to examine the relationship between the ChOLE stages and the duration of surgery.

**Results:**

This study revealed significant correlations between the ChOLE stage and respective subgroups of the classification and duration of surgery and thus complexity of procedure.

**Conclusion:**

Despite limitations, the ChOLE classification proves valuable in predicting surgical complexity and optimizing patient care. Further research is warranted to validate these findings and enhance cholesteatoma management strategies.

## Introduction

Cholesteatoma, a destructive and expanding lesion of the middle ear, poses a significant challenge in otologic surgery due to its intricate nature and potential for complications. In order to facilitate meaningful communication among healthcare professionals treating cholesteatoma, the development of a classification or staging system is imperative. Various attempts have been made to classify cholesteatoma, with commonly used staging systems including the European Academy of Neurotology and Japanese Otological Society (EAONO/JOS) classification, as well as the more recent ChOLE classification, which was introduced by Linder et al. at the International Cholesteatoma Conference in Edinburgh in 2016 [1, 2]. This classification, represented by the acronym ChOLE, stages the disease based on Ch (cholesteatoma extension), O (ossicular chain status post-surgery), L (life-threatening complications), and E (Eustachian tube function), rating them from I to IV in a numerical staging system. Allowing for a standardization of documentation and a detailed comparison of similar types of cholesteatoma extensions and assessment of treatment outcomes, guiding surgical decision-making in cholesteatoma treatment [2–5].

The selection of the surgical technique is influenced by various factors, and the localization of the cholesteatoma and surgeon’s experience play a crucial role in determining the complexity of the procedure and, consequently, the duration of the surgery [6–8]. Understanding how the ChOLE classification system correlates with operation time can be valuable for optimizing resource utilization. Particularly in healthcare systems that use the DRG (Diagnosis-related group) system, ear surgery is reimbursed relatively uniformly (and too low). Hence, it is important to show that “cholesteatoma surgery” is an extremely heterogeneous group in terms of the surgical procedures performed, the surgical effort involved and, not least, the duration of the operation. Here, classification systems could help to further optimize DRG systems in order to improve both reimbursement and resource management, provided they correlate with the necessary surgical effort and time.

Today, there exists only limited data available on the prevalence of each stage of the ChOLE classification and its practical application in clinical settings. The aim of this study was to categorize acquired cases of cholesteatoma using the ChOLE classification, considering pre- and intraoperative findings, and to delineate the prevalence of each stage. Additionally, our secondary objective was to examine the relationship between the stage and the duration of surgery, elucidating how cholesteatoma characteristics affect surgical duration and complexity. By examining this relationship, surgeons can refine their surgical approaches and allocate resources more efficiently, ultimately improving patient care in cholesteatoma cases.

## Materials and methods

Retrospectively, data from 199 consecutive patients who underwent cholesteatoma surgery at our University Hospital’s otolaryngology and head and neck surgery unit between January 2019 and March 2023 were collected and classified using the ChOLE classification system based on pre- and intraoperative findings. The ChOLE classification uses four main categories, each with up to 4 classes of severity. Single points are given for a rise in the severity level:

1. Ch - Cholesteatoma extension: Class 1 is subdivided into 1a and 1b, with 1b including extension into the sinus tympani. Class 2 involves the middle ear, extending into the attic and antrum (2a) up to the level of the lateral canal within the mastoid. Subdivision 2b entails anterior extension into the anterior epitympanum, with optional further extension into the protympanum and/or sinus tympani. Class 3 encompasses extensive bone erosion, affecting either the external ear canal or the tegmen tympani. Class 4 incorporates 4a, which is tympanomastoid cholesteatoma with infra- or supralabyrinthine, or transcochlear extensions, and 4b, an apical petrous bone cholesteatoma [2].
2. O - Ossicular status at the end of surgery: Class 0 indicates an intact and mobile ossicular chain, while Class 1 reflects erosion of the long process of the incus, with or without malleus head removal. Class 2 involves removal or erosion of the incus and stapes suprastructure while preserving a mobile footplate and malleus handle. Classes 3a and 3b refer to a mobile stapes or footplate without superstructure, and classes 4a and 4b represent a fixed stapes or footplate, respectively [2].
3. L - Life-threatening complications: L2 includes extracranial and L4 intracranial complications [2].
4. E - Eustachian tube ventilation and mastoid pneumatisation: The degree of pneumatization of the mastoid serves as an indirect sign of Eustachian tube function. E0 indicates > 50% mastoid cells aerated, E1 < 50% aerated, and E2 indicates poor pneumatisation and ventilation [2].

The overall ChOLE stage is defined as stage 1 (1–3 points), stage II (4–8 points) or stage III (8 + points) [2].

We documented patient demographics, including sex and age. Statistical analyses were conducted using IBM SPSS Statistics for Mac, version 29. Categorical data were presented as percentages, while continuous data were reported as means. One-way Anova was employed to compare independent continuous variables between the groups with p-values below 0.05 considered statistically significant.

## Results

Of the 199 patients, 88 (44.2%) were female. The mean age was 36.38 ± 23.78 years (Table 1). The majority of surgeries was conducted on the right side (53.8%). Among the cases examined, 71 (35.7%) underwent a tympanoplasty type I, 14 (7%) type II, 68 (34.2%) type IIIa (PORP), and 46 (23.1%) type IIIb (TORP). Thirty-seven patients (18.6%) were operated in a canal wall down (CWD) technique, whereas 162 patients (81.4%) were operated in canal wall up (CWU) technique. In 41 cases (20.6%) the surgery conducted was revision surgery (Table 1). The mean duration of surgery was 145.82 ± 67.45 min. Cholesteatoma surgery which resulted in a tympanoplasty type I took an average of 133.77 ± 70.42 min, type II 127.43 ± 63.87 min, type IIIa 143.60 ± 53.14 min and type IIIb 173.28 ± 76.19 min respectively. Surgery in CWU technique was shorter (135.40 ± 60.01 min) than CWD technique (191.46 ± 79.05 min). Cases of revision surgery took 166.00 ± 89.37 min (vs. primary surgery with 140.59 ± 59.74 min).

**Table 1:** Patients’ and procedural characteristics

	n (%) or mean ± SD
Total population	199
Gender (female)	88 (44.2)
Mean age (years)	36.38 ± 23.78
Right side (%)	107 (53.8)
Duration of surgery (min)	145.82 ± 67.45
Type of surgery	
Tympanoplasty type I	71 (35.7)
Tympanoplasty type II	14 (7.0)
Tympanoplasty type IIIa	68 (34.2)
Tympanoplasty type IIIb	46 (23.1)
Canal Wall Down technique	37 (18.6)
Canal Wall Up technique	162 (81.4)
Revision surgery	41 (20.6)

SD: standard deviation, min: minutes

**Fig. 1:**
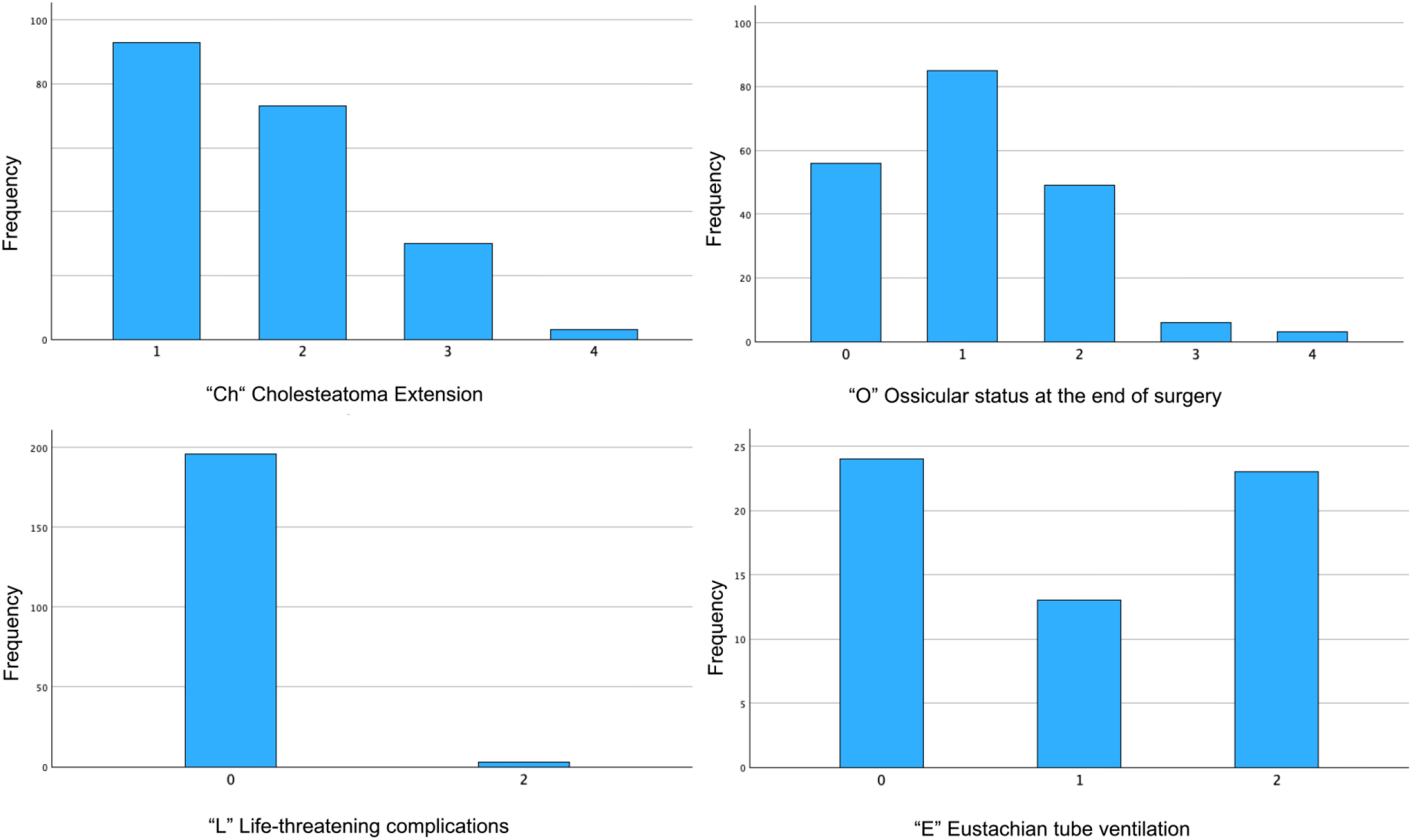
Distribution of stages

**Fig. 2:**
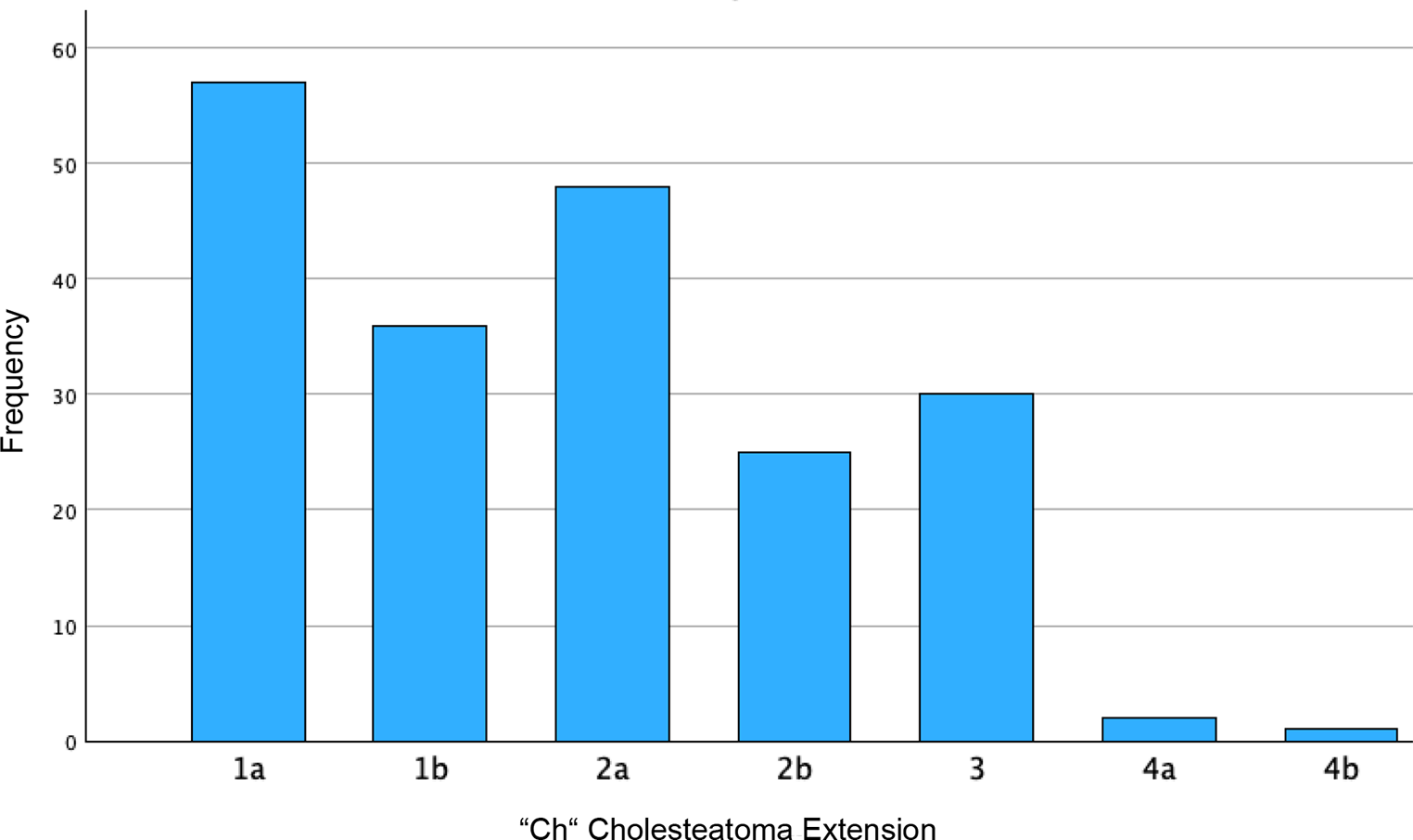
Cholesteatoma Extension

**Fig. 3:**
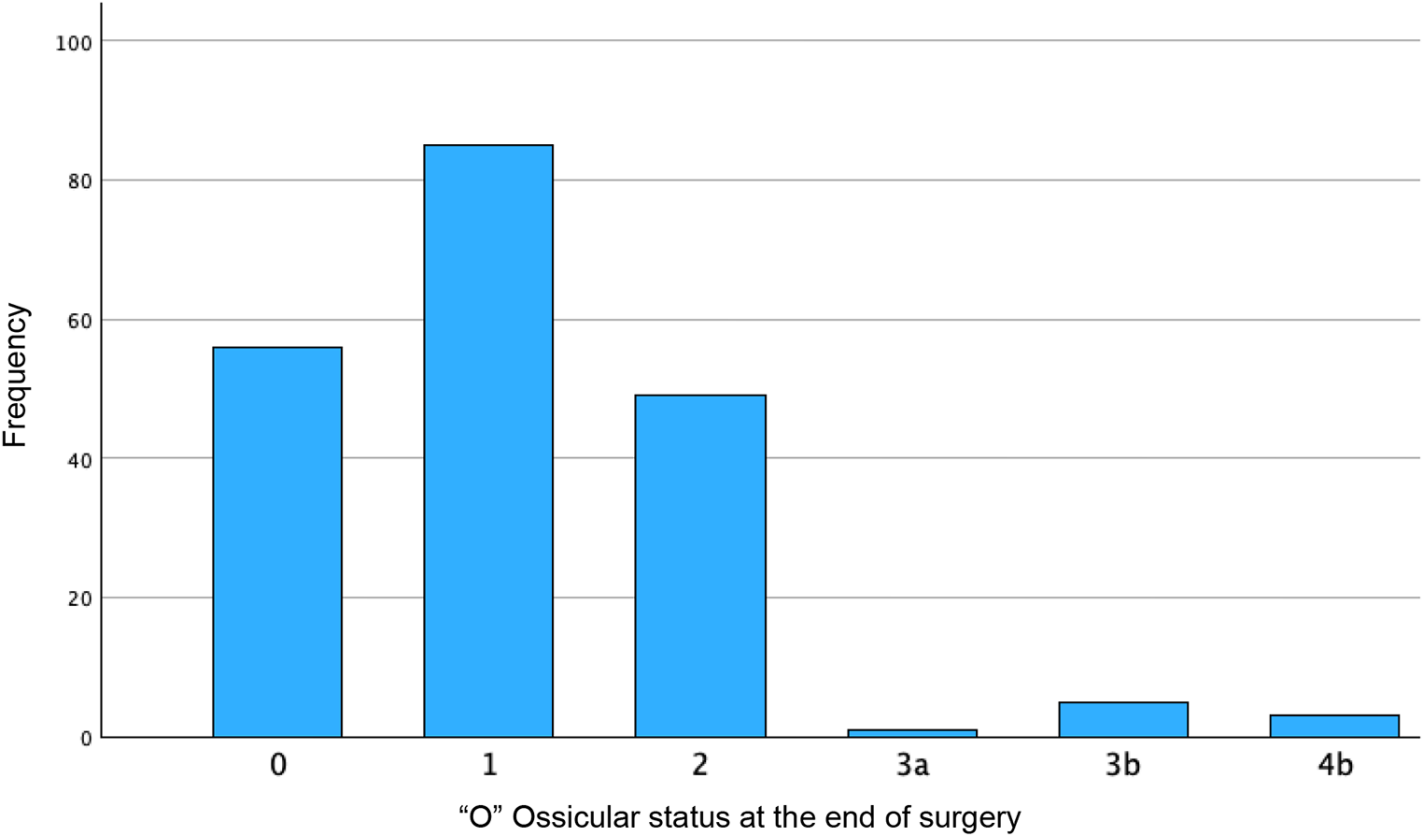
Ossicular status at the end of surgery

We categorized the patients using the ChOLE Classification System and examined the distribution of the corresponding stages (Table 2). Stage 1 was identified in 133 patients (66.8%), Stage 2 in 66 patients (33.2%), with no patients classified as stage 3 (Table 2). In terms of ChOLE classifications, 57 patients (28.6%) were classified as Ch1a, 36 patients (18.1%) as Ch1b, 48 patients (24.1%) as Ch2a, 25 patients (12.6%) as Ch2b, and 30 patients (15.1%) as Ch3. Two patients (1%) classified as Ch4a and petrous apex involvement (Ch4b) was observed in only 1 patient (0.5%) (Figs. 1 and 2). The ossicular chain status varied among the study population, with 56 patients (28.1%) having an intact ossicular chain (O0). The most common presentation was malleus and stapes present with eroded incus (O1), noted in 85 patients (42.7%). Other classifications included O2 in 49 patients (24.6%), O3a in 1 patient (0.5%), and O3b in 5 patients (2.5%). No patients were classified with fixed stapes only (O4a), while 3 patients (1.5%) had an immobile stapes footplate, leading to classification as O4b (Fig. 3). Complications were observed in 3 patients (1.5%), comprising 2 cases of facial nerve palsy and 1 case of labyrinthine fistula. No patient showed an intracranial complication (L4) (Fig. 1). The pneumatization of the mastoid was not definable in most patients (69.8%) due to missing descriptions in the operative reports or lack of imaging. Of the remaining patients, 24 (12.1%) had moderate to good pneumatization and good ventilation (E0), 13 (6.5%) had moderate to good pneumatization with poor ventilation (E1), and 23 (11.6%) were classified as E2, indicating a sclerotic mastoid (Fig. 1).

**Table 2:** ChOLE staging

	n (%)		n (%)
Ch x	0 (0.0)	**O x**	**0 (0.0)**
**Ch 1**	**93 (46.7)**	O 0	56 (28.1)
1a	57 (28.6)	**O 1**	**85 (42.7)**
1b	36 (18.1)	O 2	49 (24.6)
**Ch 2**	**73 (36.7)**	**O 3**	**6 (3.0)**
2a	48 (24.1)	3a	1 (0.5)
2b	25 (12.6)	3b	5 (2.5)
**Ch 3**	**30 (15.1)**	**O 4**	**3 (1.5)**
**Ch 4**	**3 (1.5)**	4a	0 (0.0)
4a	2 (1.0)	4b	3 (1.5)
4b	1 (0.5)		
**L x**	**0 (0.0)**	**E x**	**139 (69.8)**
L 0	196 (98.5)	E 0	24 (12.1)
**L 2**	**3 (1.5)**	**E 1**	**13 (6.5)**
L 4	0 (0.0)	E 2	23 (11.6)
**Stage I**	**133 (66.8)**		
Stage II	66 (33.2)		
**Stage III**	**0 (0.0)**		

Ch: cholesteatoma extension, O: ossicular chain status postoperative, L: life threatening complications, E: Eustachian tube function, x: not specified [1]

Our analysis revealed a significant relation between the respective subgroups of the classification and duration of surgery (Table 3). The increasing stage in the extension subgroup (Ch) correlated significantly with an increase of duration of surgery (*p* < 0.001), as did the status of the ossicular chain at the end of surgery (O) (*p* < 0.001), life threatening complications (L) (*p* = 0.018), and Eustachian tube ventilation and mastoid pneumatization (E) (*p* = 0.012). Furthermore, the overall stage correlated significantly with duration of operation (*p* < 0.001) (Table 3).

**Table 3:**
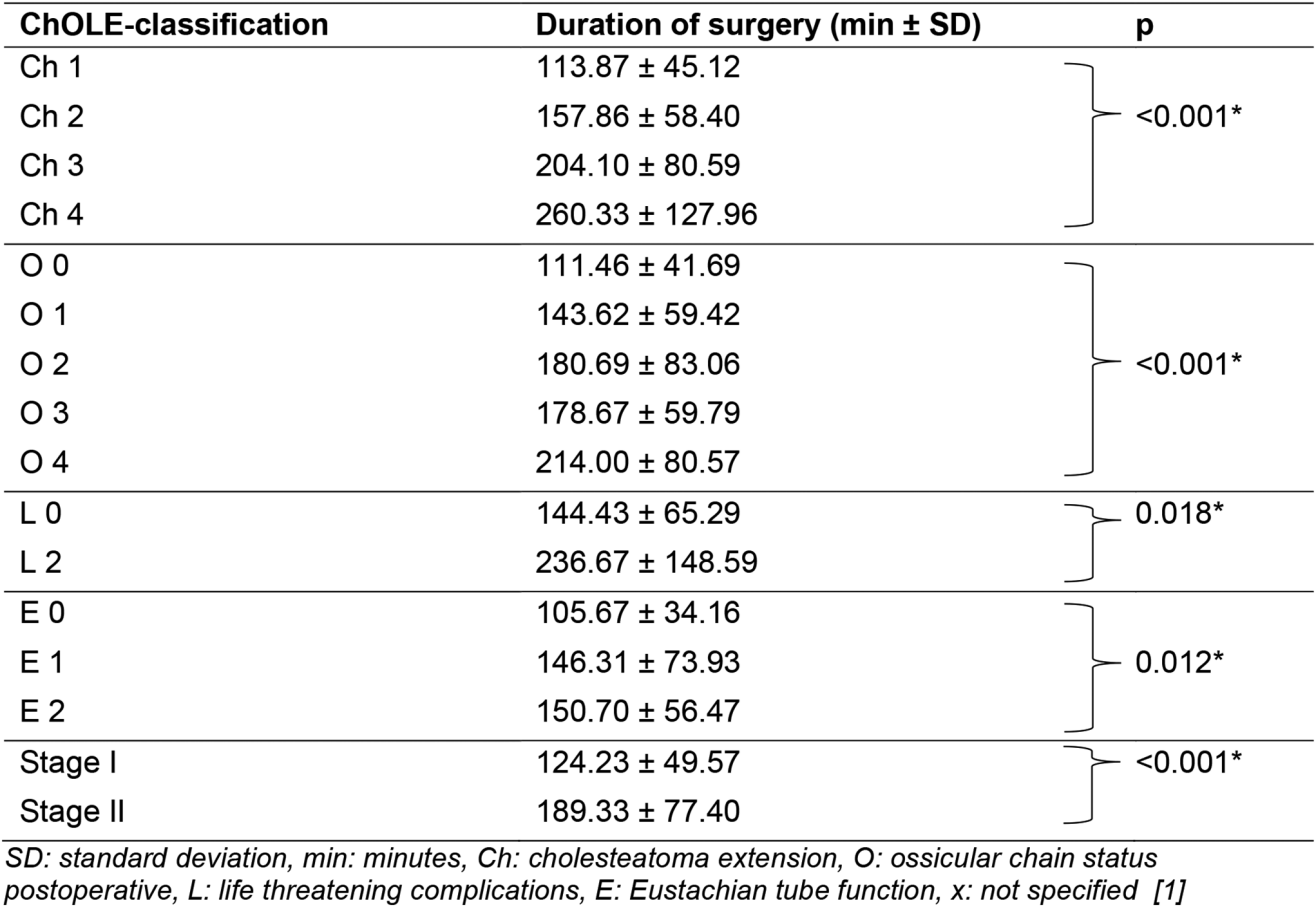
Statistical analysis

## Discussion

Our study population of 199 patients exhibited a notable distribution of cholesteatoma stages, with stage 1 being the most prevalent, followed by stage 2, and no cases classified as stage 3. Moreover, we identified a significant correlation between the stages of cholesteatoma extension (Ch) and the duration of surgery. As the stage of extension increased, so did the duration of the surgery. Furthermore, our analysis revealed significant correlations between the ossicular chain status at the end of surgery (O), life-threatening complications (L), mastoid pneumatization (E), and the duration of surgery. These findings underscore the comprehensive nature of the classification, which considers various factors influencing complexity of procedure and emphasizes the ability of the ChOLE Classification System in assessing the extent of cholesteatoma, predicting surgical complexity, and thus guiding surgical decision-making.

The distribution of subgroups within the ChOLE Classification in our study exhibits both resemblances and disparities in comparison to previous research conducted [4, 9]. In our study, stage 1 cholesteatoma was the most prevalent. However, in the studies conducted by Hajare et al. and Weiss et al., stage 2 cases were more prevalent [4, 9]. Concerning the specific ChOLE classifications, our study revealed similar proportions of Ch1a and Ch1b, and Ch2a and Ch2b cases compared to Hajare et al., suggesting comparable patterns of middle ear involvement extending to the sinus tympani across both studies. Similarly to our findings, Hajare et al. also reported Ch4 to be the least frequent subgroup among the studied population. However, Ch3 was the most frequent extension, whereas our study showed Ch1 to be the most frequent one. Weiss et al. reported a different distribution of the Ch subgroup, with stages 1 and 2 being the most frequent ones, followed by stage 4 and then stage 3, indicating potential differences in surgical techniques or patient populations [9]. In terms of ossicular chain status (O), our study found a high prevalence of malleus and stapes present with eroded incus (O1) and a low prevalence of fixed stapes (O4), consistent with findings from Weiss et al. and Hajare et al. [4, 9].

Life-threatening complications (L) were infrequent in our study, with no cases of intracranial complications (L4) described, whereas Hajare et al. described one case of a brain abscess. Hajare et al. and Weiss et al. generally reported more complications than our study [4, 9]. Regarding Eustachian tube function and mastoid pneumatization (E), our study revealed a high proportion of patients with E0 classification, indicating moderate to good pneumatization and ventilation. This finding differs from Hajare and Weiss et al., where most of the patients were classified as E2, suggesting a higher proportion of patients with sclerotic mastoids in their study population [4, 9]. Overall, while there are similarities in the distribution of some ChOLE subgroups across studies, differences exist, due to factors such as patient demographics, surgical techniques, and clinical practices on cholesteatoma classification and outcomes. Further research and comparative studies are necessary to better understand these variations and their implications for clinical management.

While the ChOLE Classification system offers a valuable framework for categorizing cholesteatomas, it is not without limitations. The classification fails to adequately represent every extent of cholesteatoma. Specifically, those that grow complicatingly medial to the ossicular chain, necessitating the degradation of individual ossicles, or those extending from the eardrum into the attic and further anteriorly into the anterior epitympanic recess or the tubal entrance, but not posteriorly toward the mastoid, are omitted. Additionally, cholesteatomas developing in the hypotympanum, particularly below the floor of the ear canal, are not accounted for.

Moreover, uncertainties exist within the “O” Category, such as the definition of a fixed stapes within stage O4a. In clinical practice, many stapes in chronic otitis media appear at least partially fixed. This distinction does not impact stages O0-O2, but it does alter stage O3a to O3b, prompting the question of why this alteration becomes relevant only when the fixed stapes is present alone, not as part of an intact ossicular chain. Furthermore, cases involving a fractured stapes footplate and rare instances where the malleus is absent but the incus is present are not addressed by the classification. Additionally, the significance of distinguishing between stages 4a and 4b is unclear, specifically regarding whether a fixed stapedial footplate still possesses a superstructure (O4a) or not (O4b). Moreover, isolated defects of the stapes superstructure, such as fractured or destroyed stapes legs due to cholesteatoma, cannot be classified.

Another critique concerns the category of “life-threatening complications” - the ‘L’ marking “life” implies severity, yet most of the listed extracranial complications are not life-threatening, particularly facial palsy, labyrinthine or semicircular canal fistulas, or labyrinthitis.

Unlike the other stages, determining the E stage is challenging to precisely define and the methodology is not entirely clear. Moreover, the distinctions between ventilation (possibly referring to “tubal function,” although gas exchange through the middle ear mucosa remains unaddressed) and pneumatization remain unclear. In such instances, classification with the ChOLE classification becomes impractical. Furthermore, the classification was partially limited due to a lack of detailed description of the respective pathology in the surgical report. In the future, aligning descriptions in the surgical reports with the classification will enhance the precision of the classification. For instance, the assessment of Eustachian tube ventilation was often unavailable as it was not documented in the surgical report, and imaging was frequently absent. A further potential limitation of this study is the retrospective design.

The ChOLE classification was primarily developed to illustrate the extent of destruction from surgical perspectives. However, it fails to depict the surgical effort involved in eradicating the cholesteatoma and especially in reconstructing the ossicular chain. Yet, these factors precisely define the temporal, technical, and financial resources required, and thus should be integral parts of a meaningful classification aimed at facilitating triage within a DRG system.

A limitation of this study is that while short-term outcomes, such as surgical duration, offer insights into immediate recovery, they may not reliably predict the long-term course of illness. Broader factors, such as patient health and the quality of follow-up care, are more critical in ensuring long-term safety. Focusing solely on surgical duration risks overlooking essential elements of recovery, as its effectiveness in warranting long-term patient safety is limited. Therefore, short-term outcomes should be considered alongside other factors to provide a more accurate assessment of long-term health outcomes.

In summary, these findings suggest that the ChOLE classification system can provide valuable insights into the complexity of cholesteatoma cases and their impact on duration of surgery. Understanding these correlations can help surgeons better anticipate surgical challenges, tailor treatment strategies, and optimize patient care in cholesteatoma management. Further research and validation of these findings could enhance surgical decision-making and improve patient outcomes in cholesteatoma surgery.

## Conclusion

The ChOLE classification system for staging cholesteatoma enables standardization in assessing the severity of the disease and reporting surgical outcomes. In conclusion, our study adds to the growing body of evidence supporting the efficacy of the ChOLE Classification System in evaluating cholesteatoma cases and predicting surgical outcomes. By providing a standardized framework for classification, this system facilitates meaningful comparisons across studies and enables informed decision-making in the management of cholesteatoma.

## Data Availability

The data cannot be shared openly due to recommendations of the ethics commission. The data can be provided upon request.
